# Low Contrast Visual Evoked Potentials for Early Detection of Optic Neuritis

**DOI:** 10.3389/fneur.2022.804395

**Published:** 2022-04-29

**Authors:** Soo-Hyun Park, Choul-Yong Park, Young Joo Shin, Kyoung Sook Jeong, Nam-Hee Kim

**Affiliations:** ^1^Department of Neurology, Department of Critical Care Medicine, Department of Internal Hospital, Inha University, Incheon, South Korea; ^2^Department of Ophthalmology, Dongguk University-Seoul Graduate School of Medicine, Dongguk University Ilsan Hospital, Goyang, South Korea; ^3^Department of Ophthalmology, Hallym University Medical Center, Seoul, South Korea; ^4^Department of Occupational and Environmental Medicine, Wonju Severance Hospital, Wonju, South Korea; ^5^Department of Neurology, Dongguk University-Seoul Graduate School of Medicine, Dongguk University Ilsan Hospital, Goyang, South Korea

**Keywords:** optic neuritis, multiple sclerosis, visual evoked potentials, visual acuity, high-contrast, low-contrast, check size

## Abstract

Optic neuritis (ON) detection is important for the early diagnosis and management of multiple sclerosis (MS) and neuromyelitis optica spectrum disorder (NMOSD). However, the conventional high-contrast visual evoked potential (VEP) used for ON detection lacks sensitivity for identifying ON presenting as mild or unremarkable visual disturbance, which is common in first-episode ON. Therefore, this study aimed to investigate whether a change in contrast or check size improves the sensitivity of VEP to first-ever ON. In total, 60 patients with the demyelinating disease (29 MS and 31 idiopathic patients with ON) without ON or with first-ever ON at least 6 months prior and 32 healthy controls underwent neuro-ophthalmic evaluations. VEPs were induced using three pattern-reversal checkerboard stimuli having, respectively, 10% contrast with a check size of 32' (LC32 VEP), 100% contrast with a check size of 32' (HC32 VEP; conventional VEP), and 100% contrast with a check size of 16' (HC16 VEP). The receiver operating characteristic (ROC) curve analysis and area under the curve (AUC) were calculated to determine the most appropriate VEP method for detecting optic nerve involvement. The optimal cut-off point was determined using the Youden index (J-index). The McNemar test was used to determine whether dichotomous proportions were equivalent. In comparison with first-ever ON eyes (*n* = 39) and healthy eyes (*n* = 64), LC32 VEP showed the highest AUC for discriminating ON (0.750, *p* < 0.001; 0.730 for HC32 VEP, *p* < 0.001; 0.702 for HC16 VEP, *p* = 0.001). In the first-ever ON group, LC32 VEP and conventional HC32 VEP were abnormal in 76.9 and 43.6%, respectively (McNemar, *p* < 0.001), and combining these tests did not improve sensitivity. These indicate that LC32 VEP is the most sensitive method for detecting first-ever ON. Visual evoked potential with 10% contrast stimuli was superior to conventional VEP for detecting first-ever ON. Thus, adding these LC stimuli might be helpful in identifying optic nerve involvement in ON with mild or unremarkable visual impairment.

## Introduction

Patients with autoimmune inflammatory diseases of the central nervous system (CNS) such as multiple sclerosis (MS) and neuromyelitis optica spectrum disorder (NMOSD) sometimes have no apparent optic neuritis (ON) despite the presence of optic nerve involvement (1, 2). ON is a crucial clinical manifestation in diagnosing MS and NMOSD (3–5). However, asymptomatic or mild ON, which are frequently seen after first-ever ON, are missed up to 65% in the measurement of visual function (6–9). Therefore, the prevalence of optic nerve involvement may be underestimated (10). However, when the optic nerve usually has substantial cumulative damage, optic nerve involvement can be easily detected by visual function tests such as visual evoked potential (VEP) (11, 12). Nevertheless, since the conventional high-contrast (HC) VEP has low sensitivity for identifying ON (8, 13), it needs to improve its sensitivity, especially for ON presenting as a mild or unremarkable visual disturbance. Because early recognition of optic nerve involvement can accelerate the diagnosis of MS or NMOSD and initiate appropriate treatment critical to the patient's quality of life (14).

Optic nerve lesions were either substantiated clinically or paraclinically by magnetic resonance imaging (MRI), VEP, or optical coherence tomography (OCT). Still, diagnostic sensitivity and specificity of these tests were insufficient to support incorporation into the McDonald criteria (4). VEP is superior to MRI for evaluating the visual function, such as cost-effective aspects, and provides an integrated assessment of the visual pathway (15, 16). In addition, VEP can present prolonged latency in prechiasmal and retrochiasmal involvement of the visual pathway and has been used as a paraclinical test to diagnose MS and NMOSD (16, 17). Since the diagnosis of ON was often missed, previous studies have attempted to increase the detection rate of optic nerve involvement (8, 9, 15, 17–19). Recently, the inter-eye differences thresholds by OCT were suggested to be useful in identifying the presence of asymptomatic optic nerve involvement for the MS diagnostic criteria (11, 20).

Furthermore, it has been reported that low-contrast (LC) stimuli increase the sensitivity of VEP to optic nerve involvement in the early stage of diseases (19, 21, 22). In addition, we previously found that LC visual acuity (VA) measurements can detect visual disturbances in ON than HCVA (23). Thus, the aim of this study was to investigate whether LCVEP could improve VEP sensitivity in ON and mainly focused on identifying optic nerve involvement with mild or unremarkable visual impairment in first-ever ON.

## Materials and Methods

### Study Design and Population

This study was conducted using a retrospective survey of medical records between January 2013 and December 2016. All patients with the clinical impression of ON, MS, or NMOSD, which was assessed by both neurologist (NH K) and ophthalmologist (CY P), underwent serologic evaluation such as astrocyte water channel aquaporin-4 (AQP4-IgG) antibodies, imaging studies including brain or orbital MRI, and ophthalmological evaluations including VEPs adding LC stimulation and smaller check size. We investigated 120 eyes with demyelinating diseases (29 patients with MS who met the 2010 McDonald criteria and 31 patients with idiopathic ON) and 64 eyes of 32 healthy controls (24–26). For eyes with ON, we included only those with first-ever ON that occurred at least 6 months before minimizing the acute effect of inflammation (16) and ensuring ON's evaluation with mild or unremarkable visual impairment. Additionally, we evaluate the AQP4-IgG to idiopathic ON and ON in the setting of MS in all patients (27). Subjects were excluded from analysis, if they had a history of glaucoma, diabetes, or retinal disease, affecting visual function. This study was approved by the local Human Research Protection Office/Institutional Review Board, and patients or their legally authorized representatives provided written informed consent.

### Visual Evoked Potential

Visual evoked potentials were elicited in response to 2-Hz pattern-reversal checkerboard stimuli generated by a Medelec Synergy Visual Electrodiagnostic Testing System (Oxford Instrument Co., Surrey, UK), with an active electrode fixed on the scalp at the Oz position and referenced to Fz (28). We studied the responses to stimuli with 100% contrast (HC) and 10% contrast (LC) at check size of 32′ min of arc and 100% contrast at check size of 16′ min of arc. The output signals of the electrodes were amplified and passed through a bandpass filter with low and high cut-off frequencies of 1–100 Hz. If the waveform was unobtainable, a value of 250 ms was used, representing the most delayed waveform obtainable.

### Visual Acuity

All of the visual tests were performed monocularly. The standard Snellen chart measured the best-corrected conventional VA with 100% HCVA. For LCVA, the 2.5% LC Sloan letter charts were used. VA was expressed using a decimal scale but was transformed to the logarithm of the minimum angle of resolution (LogMAR) for statistical analysis.

### Statistical Analysis

Data are presented as mean (standard deviation), min, max, median (interquartile range), number (percentage), or percentile (25th, 50th, and 75th) as appropriate. Comparisons between groups were performed using the Student *t*-test or Mann–Whitney *U*-test, considering normality and the properties of the variables. A multivariate analysis of variance (MANOVA) compared three groups on two dependent measures, age and gender. Receiver operating characteristic (ROC) curve analysis and the area under the curve (AUC) were used to determine the most sensitive VEP method for detecting first-ever ON.

Based on the Youden index (J-index), the optimal cut-off point was determined as the point with the best sensitivity-specificity balance in comparing the control and ON groups (29). This point was then established as the reference value for each type of stimulus. The McNemar test determined whether dichotomous proportions were equivalent between VEP methods. Pearson's correlation coefficient or partial correlation coefficient was used to assess correlations among visual function measurements. All statistical analyses were performed using SPSS 26.0 for Windows (SPSS Inc., Chicago, IL, USA). Statistical significance was set at *p* < 0.05.

## Results

The characteristics of each group are shown in Table 1. In total, 64 healthy eyes, 39 eyes with first-ever ON, and 81 eyes without ON (non-ON) were assessed. In the first-ever ON, the mean VA was 0.8 decimal (Table 2), which corresponds to unremarkable visual impairment based on the World Health Organization (WHO) definition (30). WHO defines remarkable visual impairment as less than 0.33 decimal (30).

**Table 1:** Baseline characteristics according to study groups^a^.

	**Control** **(*n* = 64 eyes)**	**Non-ON** **(*n* = 81 eyes)**	**First-ever ON** **(*n* = 39 eyes)**
Age (mean ± SD, year)^b, c^	47.2 ± 15.6	39.9 ±12.5	41.8 ± 15.9
Gender, female (*n*, %)	28 (43.8)	48 (59.3)	22 (56.4)
**Diagnosis**			
MS (*n*, %)	0 (0)	49 (62.0)	7 (17.9)
ON (*n*, %)	0 (0)	30 (38.0)	32 (82.1)
Time from ON attack (median [IQR], months)	–	9.9 (6.0–13.0)	10.8 (6.0–16.0)
Bilateral ON (*n*, %)	–	–	16 (41.0)
EDSS, mean ± SD	–	2.7 ± 2.0	3.7 ± 2.0

*^a^Adjusted age, gender.*

*^b^Control vs. Non-ON, p = 0.653.*

*^c^Control vs. First-ever ON, p = 0.306.*

*ON, optic neuritis; Non-ON, non-optic neuritis; MS, multiple sclerosis; SD, standard deviation; EDSS, expanded disability status scale*.

**Table 2:** The value of Visual acuity and VEP latency between high-contrast and low-contrast.

	**Mean /Median**	**Min**	**Max**	**Percentile**			***p*-value**
				**25th**	**50th**	**75th**	
**HCVA, LogMAR (Decimal) (Median)**							
Control	0.00 (1.00)	−0.18 (1.53)	0.70 (0.20)	0.00 (1.00)	0.00 (1.00)	0.15 (0.70)	0.878*
Non-ON	0.00 (1.00)	−0.18 (1.53)	0.70 (0.20)	−0.40 (2.50)	0.00 (1.00)	0.15 (0.70)	0.003**
First-ever ON	0.10 (0.80)	−0.30 (2.00)	2.30 (0.005)	0.00 (1.00)	0.10 (0.80)	0.52 (0.30)	0.007***
**LCVA, LogMAR (Decimal) (Median)**							
Control	0.70 (0.20)	0.40 (0.40)	0.90 (0.125)	0.50 (0.32)	0.70 (0.20)	0.80 (0.16)	0.306*
Non-ON	0.80 (0.16)	0.40 (0.40)	2.30 (0.005)	0.55	0.70 (0.20)	0.80 (0.16)	0.002**
First-ever ON	0.70 (0.20)	0.30 (0.50)	2.30 (0.005)	0.70 (0.20)	0.80 (0.16)	2.30 (0.005)	<0.001***
**HC32 VEP latency, ms (Mean)**							
Control	100.54	86.75	127.25	93.81	100.75	104.69	0.494*
Non-ON	102.27	88.75	133.25	96.25	101.50	104.75	<0.001**
First-ever ON	110.26	86.25	250.00	100.25	106.75	127.25	<0.001***
**LC32 VEP latency, ms (Mean)**							
Control	117.30	85.75	168.75	109.88	116.13	124.06	0.072*
Non-ON	120.92	99.75	162.25	113.75	120.75	124.38	<0.001**
First-ever ON	127.89	109.50	250.00	119.50	126.50	149.50	<0.001***
**HC16 VEP latency, ms (Mean)**							
Control	107.77	93.25	137.50	99.50	106.75	113.69	0.417*
Non-ON	110.23	92.75	147.25	102.38	107.75	115.00	<0.001**
First-ever ON	117.99	96.25	250.50	103.75	117.00	142.00	<0.001***

*Control vs. non-ON; **Non-ON vs. 1st ON; ***Control vs. 1st ON.

*VEP, visual evoked potential; ON, optic neuritis; Non-ON, non-optic neuritis; HC, high-contrast; LC, low-contrast*.

**Figure 1:**
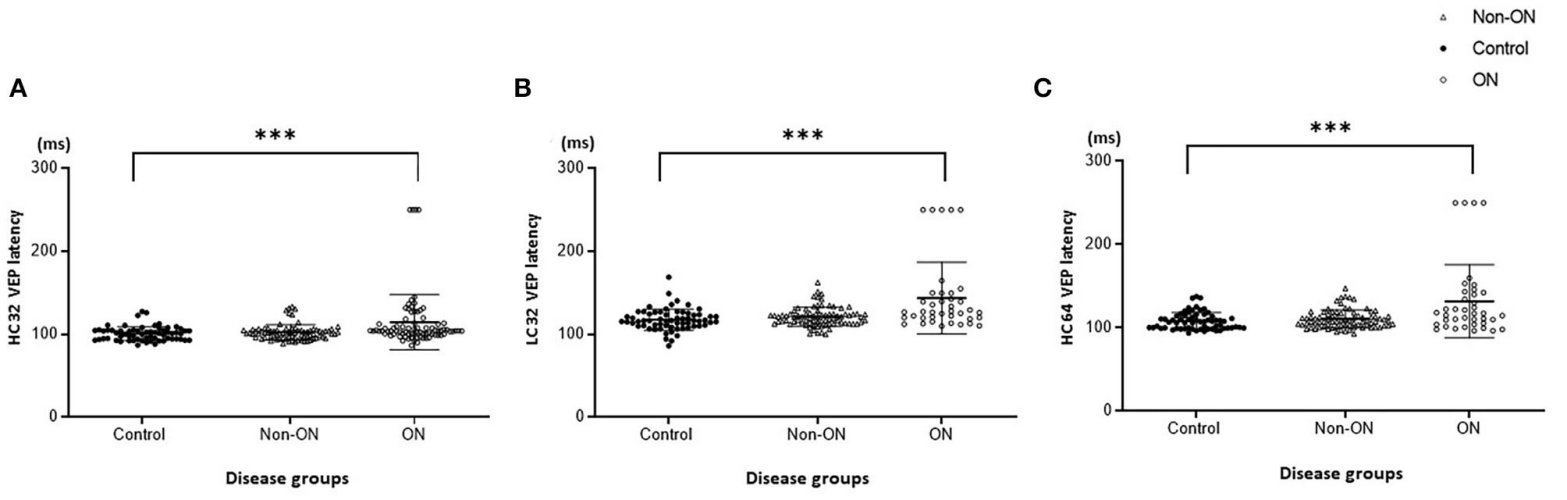
Visual evoked potential (VEP) latency with high-contrast (HC, 100%) and low-contrast (LC, 10%) stimulation. **(A)** HC32 VEP for patients without optic neuritis (ON) or with first-ever ON and healthy controls. **(B)** LC32 VEP for patients without ON or with first-ever ON and healthy controls. **(C)** HC16 VEP for patients without ON or with first-ever ON and healthy controls. ***p < 0.001.

**Figure 2:**
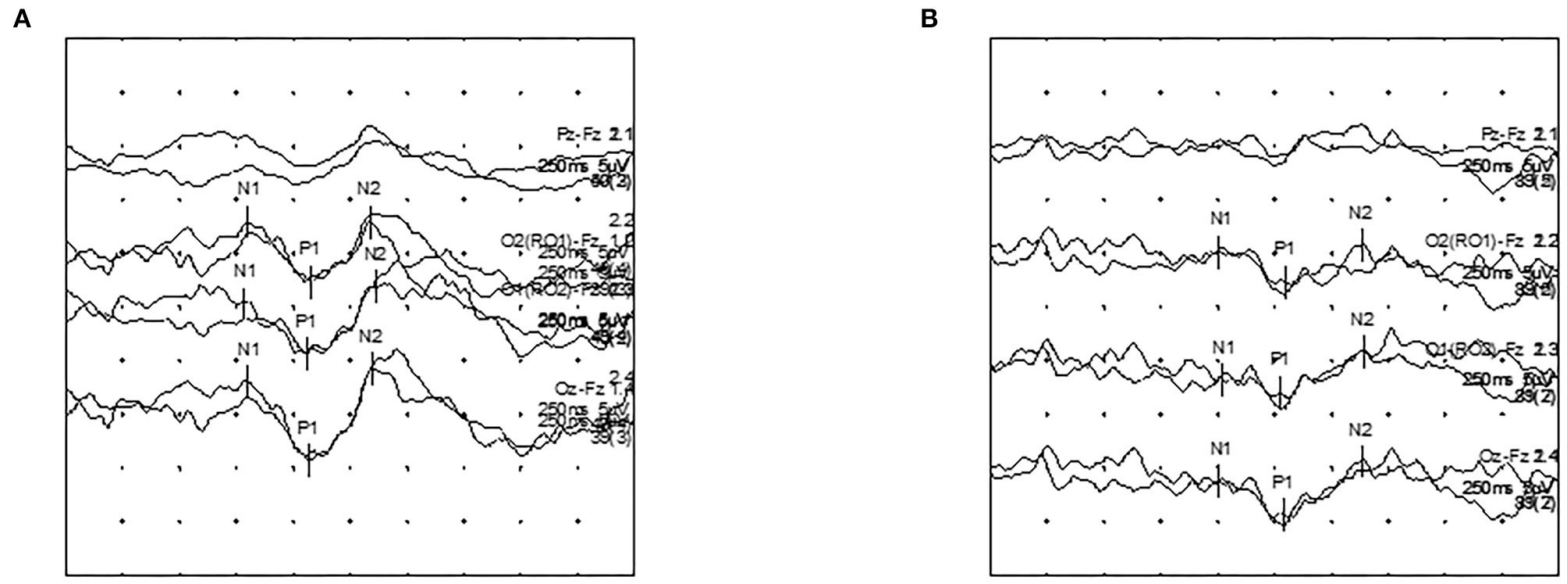
Visual evoked potential (VEP) latency recordings according to contrast stimuli in a first-ever ON patient. **(A)** HC32 VEP. **(B)** LC32 VEP.

The VEP findings with LC32', HC32', and HC16' stimuli are shown in Figure 1 and Table 2. Latencies were proportionally prolonged against HC stimuli with 16' check size and LC32' check size in each group, similar to the conventional stimulus with HC32' check size in Figure 2. In addition, the latency of the first-ever ON group was significantly prolonged than in the non-ON or control group (*p* < 0.05 in each). However, there was no significant difference in the latency between the control and non-ON groups.

**Figure 3:**
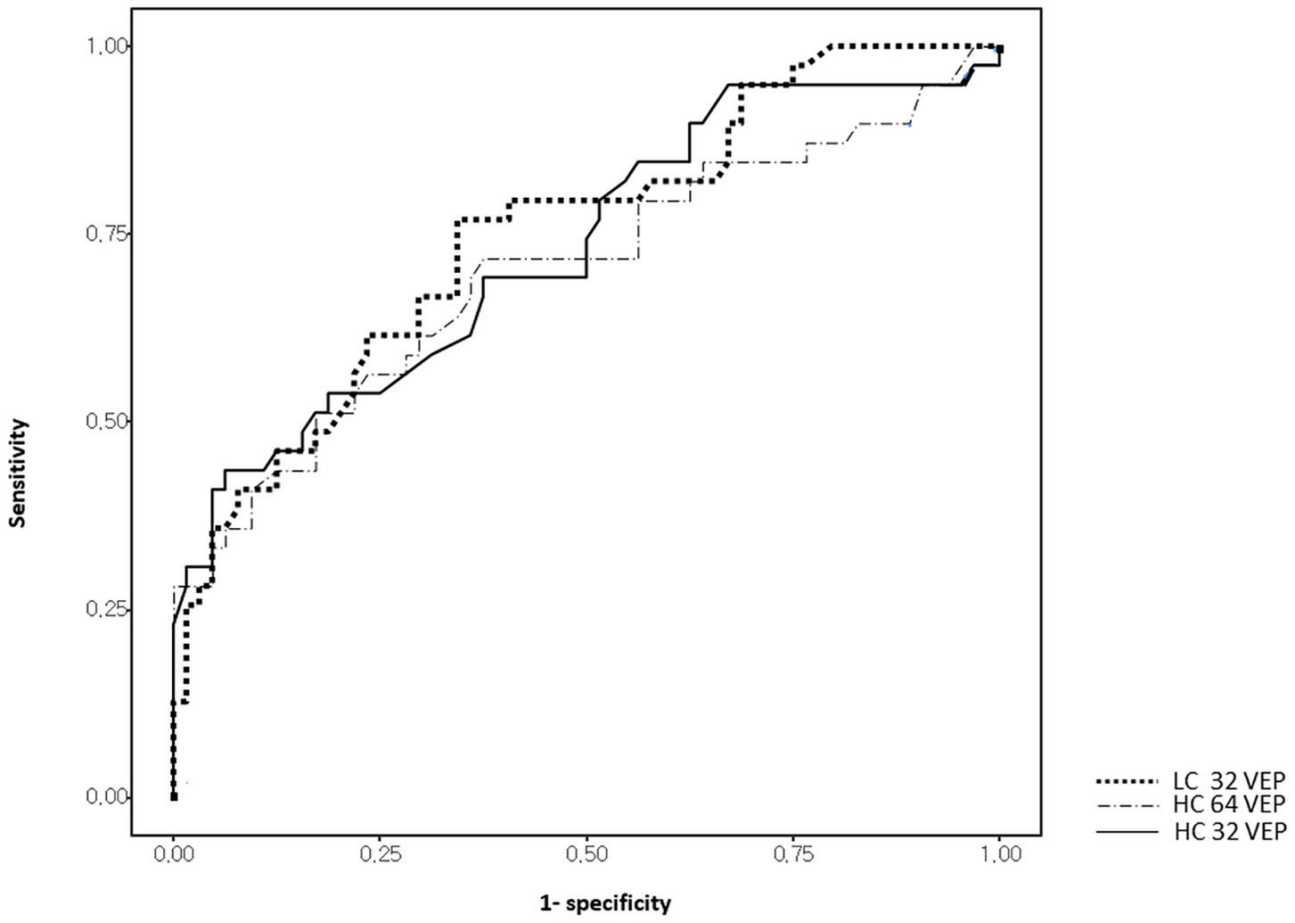
Receiver operating characteristic (ROC) curves of visual measures for discrimination between first-episode optic neuritis (ON) and controls.

Receiver operating characteristics curve analyses of visual tests are shown in Figure 3 and Table 3. The optimal cut-off points were obtained and established as reference values for each stimulus as 111.5 ms for HC32 VEP, 119.4 ms for LC32 VEP, and 109.1 ms for HC16 VEP. In comparison with first-ever ON eyes (*n* = 39) and healthy eyes (*n* = 64), LC32 VEP showed the highest AUC (0.750, *p* < 0.001) for discriminating ON compared to other VEP settings (0.73 for HC32 VEP, *p* < 0.001; 0.702 for HC16 VEP, *p* = 0.001). In addition, the LC32 and HC32 VEPs were abnormal in 76.9% and 43.6% of eyes in the first-ever ON group, respectively (McNemar, *p* < 0.001). The combination of HC32 VEP and LC32 VEP did not provide higher sensitivity (76.9%) when compared with LC32 VEP alone (McNemar, *p* < 0.001). In the non-ON group, LC32 and HC32 VEPs were abnormal in 53.1 and 9.9% of eyes, respectively (McNemar, *p* < 0.001), and the combination of the two tests also did not improve sensitivity. In the control group, LC32 and HC32 VEPs were abnormal in 34.4 and 6.3% of eyes, respectively (McNemar, *p* < 0.001). The combination of the two tests did not improve sensitivity. Considering these results, LC32 VEP was the most sensitive method for detecting optic nerve involvement with unremarkable visual impairment compared with HCVEP.

**Table 3:** Receiver operating characteristic curve analysis of visual functions to discriminate between First-ever ON and Controls.

	**AUC (95% *CI*)**	***p-*value**	**Cut-off value**	**Sensitivity (%)**	**Specificity (%)**	**J-index**
HCVA LogMAR	0.625 (0.507–0.743)	0.148	0.30	38.5	87.5	0.260
LCVA LogMAR	0.669 (0.517–0.821)	0.009	1.80	30.4	100.0	0.304
HC32 VEP	0.730 (0.626–0.833)	<0.001	111.50	43.6	93.8	0.373
LC32 VEP	0.750 (0.653–0.847)	<0.001	119.38	76.9	65.6	0.425
HC16 VEP	0.702 (0.591–0.812)	0.001	109.13	71.8	62.5	0.343

*HCVA, high-contrast visual acuity; LCVA, low-contrast visual acuity; VEP, visual evoked potential; ON, optic neuritis; AUC, area under the receiver operating characteristic curve; CI, confidence interval*.

**Figure 4:**
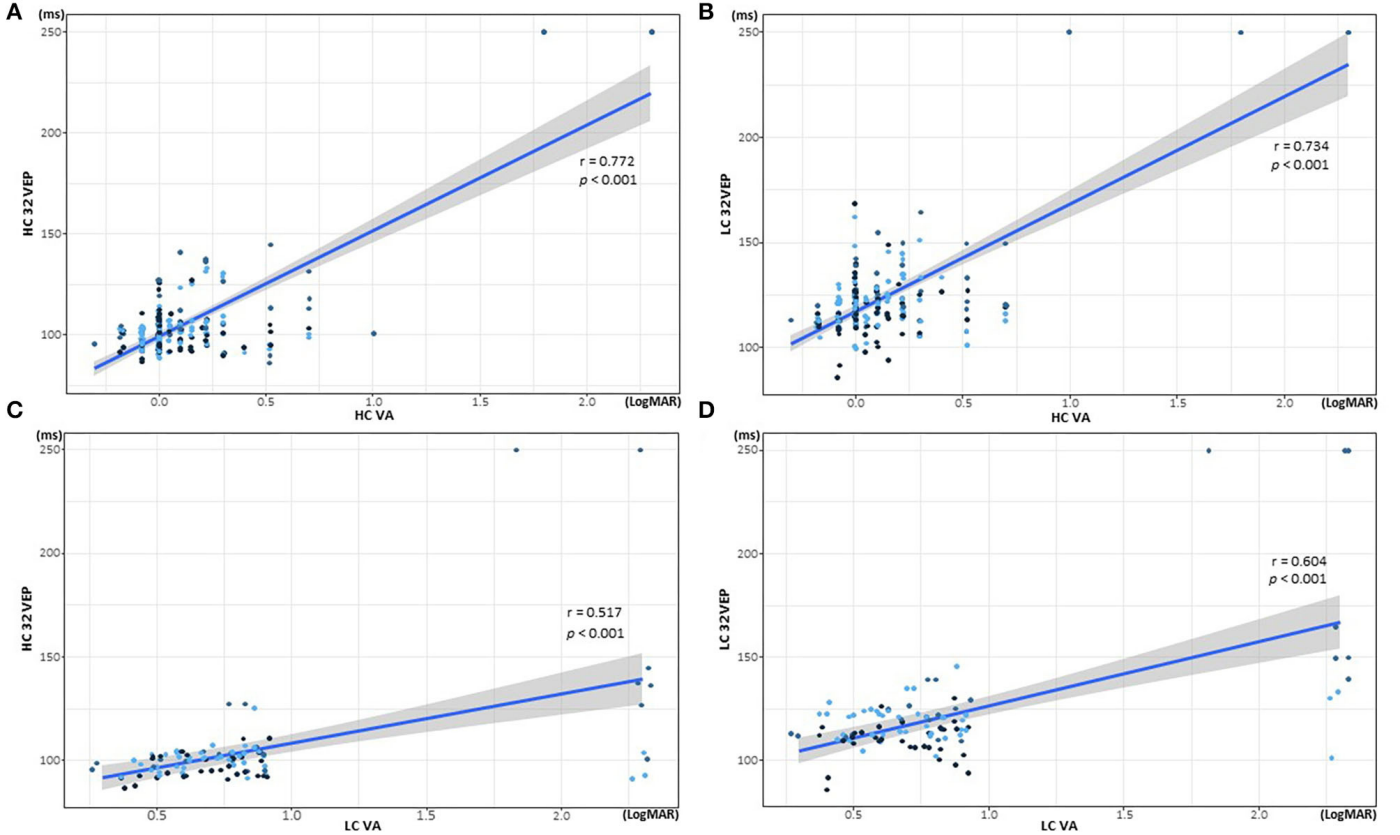
Correlations of visual acuity (VA) and visual evoked potential (VEP) latency according to contrast stimuli. **(A)** Correlation of high-contrast (HC) 32 VEP and HCVA. **(B)** Correlation of low-contrast (LC) 32 VEP and HCVA. **(C)** Correlation of HC32 VEP and LCVA. **(D)** Correlation of LC32 VEP and LCVA.

In the correlation analysis (Figure 4 and Table 4), all VEP methods were correlated with VAs. HCVA had a stronger correlation with each VEP method (HC32 VEP, *r* = 0.772, *p* < 0.001; LC32 VEP, *r* = 0.734, *p* < 0.001) compared with LCVA (HC32 VEP, *r* = 0.517, *p* < 0.001; LC32 VEP, *r* = 0.604, *p* < 0.001). LCVA was more correlated with LC32 VEP (*r* = 0.577, *p* < 0.001) than with HC32 VEP (*r* = 0.491, *p* < 0.001).

**Table 4:** Correlation between VA and VEP latency according to contrast and check size.

	**HC 32 VEP**	**LC 32 VEP**	**HC 64 VEP**
HCVA	*r* = 0.772, *p* <0.001	*r* = 0.734, *p* <0.001	*r* = 0.787, *p < * 0.001
LCVA	*r* = 0.517, *p* <0.001	*r* = 0.604, *p* <0.001	*r* = 0.602, *p < * 0.001

*HCVA, high-contrast visual acuity; LCVA, low-contrast visual acuity; VEP, visual evoked potential*.

## Discussion

This study investigated whether LC stimulation could improve VEP sensitivity, especially to the first-ever ON with mild visual impairment. We revealed that LC32 VEP is superior to conventional HC32 VEP or HC16 VEP for detecting first-ever ON. Furthermore, in eyes with first-ever ON, VEP with LC stimuli detected more abnormalities (76.9%) than conventional VEP with HC stimuli (43.6%). Furthermore, in subclinical optic nerve involvement detection, VEP with LC stimuli detected abnormalities in 53.1%, while conventional VEP with HC stimuli detected abnormalities in 9.9%. These findings imply that VEP with LC stimulation might be more sensitive than conventional HCVEP to detect ON without remarkable visual impairment.

Early detection of ON is crucial for achieving better disease outcomes and recovery (31). Therefore, several diagnostic methods for early detection of ON have been evaluated (1, 12, 15, 18–20, 32, 33). VEP is more widely used in diagnostic procedures than MRI due to its non-invasiveness, simplicity, and ability to reflect dynamic changes in CNS involvement (34, 35). In clinical practice, when optic nerve damage is suspected, VEP testing can help capture the prior demyelinating injury (36). Prolongation of VEP latency indicates demyelination, and the degree of latency delay correlates with the magnitude of the demyelinated area in animal models (37). Furthermore, VEP amplitude was correlated with axonal damage in animal models (37).

Visual evoked potentials have been used to support the diagnosis of ON by providing evidence of demyelinating lesions within the visual pathway (3, 38, 39). Abnormal VEP measures are found in 37–55% of patients with MS (20, 37). In our previous study of ON with NMOSD, which usually presents with more severe impairment than does ON with MS, abnormal VEP measures were less frequent in first-ever ON than in eyes with two or more episodes of ON (67 vs. 83%) (20). Conventional VEP detected abnormalities in 67–81% of definite ON but only detected abnormalities in 24–40% of subclinical optic nerve involvement (20, 40, 41). After the first episode or in the early stage of the disease, patients with ON complain of “some discomfort” in their vision but present normal values in conventional VEP (19).

Given that conventional VEPs have lower sensitivity for detecting subtle involvements of visual function in mild or first-ever ON (20, 40, 41), we investigated whether lower contrast visual stimuli may improve the sensitivity of VEP in identifying first-ever ON. Previous studies have demonstrated that LCVA is more sensitive for ON discrimination than HCVA (19, 28). However, there have been controversies regarding improving VEP sensitivity by LC stimuli in patients with MS (15, 18). In a study of 15 patients with MS and 15 controls using 10- and 100%-contrast VEPs, VEP latencies were increased or absent in response to LC stimuli in the MS group, proving that LCVEP is more helpful in identifying demyelinating optic neuropathy than HCVEP (15). In contrast, another study of 23 patients with MS and 19 controls demonstrated that LCVEP is not superior to conventional HCVEP and may not have better sensitivity for the early detection of optic demyelination, owing to increased variations of the evoked waveforms when LC stimuli are used (18). Furthermore, the sensitivity of LCVEP has not been estimated in the previous studies due to the small number of patients.

This study of 60 patients with demyelinating disease and 32 controls revealed that LCVEP has higher sensitivity and is thus more useful for detecting ON without remarkable visual impairment than HCVEP. In addition, we established the normal values of LC32 VEP (≤ 119.4 ms) and HC16 VEP (≤ 109.1 ms) for detecting optic nerve abnormality. VEP latency with LC stimuli was more significantly correlated with LCVA than with HCVA, suggesting that the visual pathway of LCVA is more related to the pathway of LCVEP. LC stimuli may be clinically helpful for detecting early optic abnormalities. This study adds to the evidence that changing VEP settings can improve the detection of unremarkable visual dysfunction in demyelinating diseases.

Visual evoked potentials are affected by the various stimulus settings, such as the check size, luminance, contrast, and spatial frequency (40). Significantly, the lower contrast and a smaller check size than conventional stimuli can delay conduction through different visual pathways (41). The visual pathway consists of two major parallel retinocortical pathways, the magnocellular and the parvocellular pathways (22, 42, 43). The magnocellular and parvocellular visual pathways consist of disparate retinal ganglion cells, have different layers in the lateral geniculate nucleus, and end in separate input layers in the primary visual cortex (44). The LC and HC stimulations are related to different visual pathways (43). Pattern reversal VEPs in the conventional HC are mainly related to the parvocellular pathway, which originates in the midget ganglion cells of the retina, composed of neurons with short axons and low-speed transmission (22, 36, 43–45). The magnocellular pathway, which originated in the parasol ganglion cells of the retina and consists of neurons with long axons and high-speed transmission, is primarily involved in the response to LCVEP stimulation (38, 43, 45–47). VEP amplitude tends to increase with smaller checks, but VEP latency does not change (48, 49). While the magnocellular pathways are responsive to larger check size, the parvocellular pathway is more responsive to smaller checks (38, 45, 46). Conventional VEP is primarily a reflection of activity originating in the central 2–6 degrees of the visual field corresponding to the central retina using appropriate check size (28). Since most (up to 95%) of the receptors in the central retina are connected to midget ganglion cells, conventional VEPs are mainly involved with the parvocellular pathway (43, 50).

This study had several limitations. We did not include patients with NMOSD, which frequently present with ON. Because ON eyes in NMOSD usually have more severe visual impairment than ON in MS, abnormalities in ON are already easily detected by conventional VEP (7, 8, 15, 41). However, there is a need for further evaluation using LCVEP in the first-ever ON of NMOSD with relatively mild visual impairment. A recent report for VEP in NMOSD suggested progressive VEP latency delay occurring independently of acute ON, which requires further prospective longitudinal studies for the clinical relevance (51). In this study, AQP4-IgG, which was only available in the study period, was identified without checking MOG-IgG. Since MOG-IgG was found in 4% of a prospective study of ON (27), further investigation for VEP in MOG-IgG positive ON may be needed. This study focused that first-ever ON had occurred at least 6 months prior for minimizing the acute effect of inflammation. Therefore, steroid treatment was not assessed because VEPs were not done in acute ON. Further study of the effect of steroids or disease-modifying agents on VEPs may be required. Although LCVEP has markedly higher sensitivities for detecting ON, abnormalities sometimes appear in control eyes. In this study, half of the non-ON eyes had abnormalities, while one-third of the controls also had abnormalities in LCVEP. Previous studies reported that VEP with 10% contrast stimulation was absent in about 30% of patients with clinically defined MS and 10% of normal controls (15). Absent or delayed P100 waveforms are more frequent in LCVEP, which is a profound disadvantage in evaluating optic conditions (18). Since more abnormalities were detected in non-ON eyes than in control eyes, and LCVEP also detected all abnormalities detected by conventional VEP, it could also be concluded that the high sensitivity of LCVEP may be particularly useful for finding objective evidence of ON without remarkable visual impairment in the early stage of MS. LCVEP could be used in “Remyelination trials” as an additional monitoring test to measure the degree of remyelination by measuring optic nerve conduction at the stage of improvement by remyelination (52).

In conclusion, VEP using LC stimuli is superior to conventional VEP for the diagnosis of first-ever ON. For patients with mild visual impairment or with normal conventional HCVEP, LCVEP can be performed as an additional test to confirm optic nerve involvement. This result may improve the clinical sensitivity of conventional modalities for the early detection of optic nerve abnormalities. Therefore, in ON with mild or unremarkable visual impairment, adding VEP with LC stimuli might be helpful in detecting optic nerve involvement for the early diagnosis of demyelinating diseases.

## Data Availability Statement

The raw data supporting the conclusions of this article will be made available by the authors, without undue reservation.

## Author Contributions

S-HP, C-YP, YS, KJ, and N-HK: conception and organization of the research project. S-HP, C-YP, YS, and N-HK: execution of the research project. S-HP, KJ, and N-HK: analysis and interpretation and review and critique of the manuscript. S-HP and N-HK: writing of the first draft of the manuscript. All authors contributed to the article and approved the submitted version.

## Funding

This work was supported by the National Research Foundation (NRF) of Korea (Grant Number 2021R1F1A1049320). This work was supported by Dongguk University Ilsan Hospital (Grant Number 2018-2109-00).

## Conflict of Interest

The authors declare that the research was conducted in the absence of any commercial or financial relationships that could be construed as a potential conflict of interest.

## Publisher's Note

All claims expressed in this article are solely those of the authors and do not necessarily represent those of their affiliated organizations, or those of the publisher, the editors and the reviewers. Any product that may be evaluated in this article, or claim that may be made by its manufacturer, is not guaranteed or endorsed by the publisher.
